# A Review on Plant-Mediated Synthesis of Bimetallic Nanoparticles, Characterisation and Their Biological Applications

**DOI:** 10.3390/ma14247677

**Published:** 2021-12-13

**Authors:** Lavinia Berta, Năstaca-Alina Coman, Aura Rusu, Corneliu Tanase

**Affiliations:** 1 Department of General and Inorganic Chemistry, “George Emil Palade” University of Medicine, Pharmacy, Sciences and Technology of Târgu Mureș, 38 Gheorghe Marinescu Street, 540139 Târgu Mureș, Romania; lavinia.berta@umfst.ro; 2 Medicine and Pharmacy Doctoral School, George Emil Palade University of Medicine, Pharmacy, Science and Technology of Târgu Mureș, 540142 Târgu Mureș, Romania; nastaca-alina.coman@umfst.ro; 3 Pharmaceutical and Therapeutical Chemistry Department, Faculty of Pharmacy, George Emil Palade University of Medicine, Pharmacy, Science, and Technology of Targu Mures, 540142 Târgu Mureș, Romania; 4 Pharmaceutical Botany Department, Faculty of Pharmacy, George Emil Palade University of Medicine, Pharmacy, Science and Technology of Târgu Mureș, 540142 Târgu Mureș, Romania; corneliu.tanase@umfst.ro

**Keywords:** bimetallic nanoparticles (BNPs), green synthesis, phytochemicals, polyphenols, antimicrobial, anticancer

## Abstract

The study of bimetallic nanoparticles (BNPs) has constantly been expanding, especially in the last decade. The biosynthesis of BNPs mediated by natural extracts is simple, low-cost, and safe for the environment. Plant extracts contain phenolic compounds that act as reducing agents (flavonoids, terpenoids, tannins, and alkaloids) and stabilising ligands moieties (carbonyl, carboxyl, and amine groups), useful in the green synthesis of nanoparticles (NPs), and are free of toxic by-products. Noble bimetallic NPs (containing silver, gold, platinum, and palladium) have potential for biomedical applications due to their safety, stability in the biological environment, and low toxicity. They substantially impact human health (applications in medicine and pharmacy) due to the proven biological effects (catalytic, antioxidant, antibacterial, antidiabetic, antitumor, hepatoprotective, and regenerative activity). To the best of our knowledge, there are no review papers in the literature on the synthesis and characterisation of plant-mediated BNPs and their pharmacological potential. Thus, an effort has been made to provide a clear perspective on the synthesis of BNPs and the antioxidant, antibacterial, anticancer, antidiabetic, and size/shape-dependent applications of BNPs. Furthermore, we discussed the factors that influence BNPs biosyntheses such as pH, temperature, time, metal ion concentration, and plant extract.

## 1. Introduction

The study of nanoparticles (NPs) is of great interest in research and technology, being a constantly expanding field, especially in the last decade, having been studied due to size-dependent physical and chemical properties [1]. Nanotechnology has attracted the attention of researchers due to its many application possibilities, with NP already being used in various biomedical fields (chemotherapy, diagnostics, biomedical devices, drug delivery systems, cosmetics, etc.) [2], but also in the chemical [3], energy [4], electronics [5], and space [6] domains. NPs biosynthesis using natural extracts has many advantages, being preferred by researchers, to the detriment of chemical synthesis [7,8,9]. Green synthesis is simple, economical, and safe for the environment. In chemical synthesis, the products obtained are unstable, requiring stabilising and protective agents, which are generally toxic and expensive [10,11].

Among them all, the most requested are the monometallic and bimetallic nanoparticles (BNPs) of noble metals (Au, Ag), which are efficient in various industries [12,13]. BNPs are of greater interest than monometallic NPs because they have better optical, electronic, magnetic, catalytic, and medical applications; this is due to special mixing patterns and synergistic effects (between the two metallic NPs that form BNPs) [10,14,15]. Furthermore, surface plasmon resonance (SPR) characteristics can be tuned systematically from 520 to 400 nm by changing the Au–Ag BNP alloy composition. The plasmonic properties of the alloy nanomaterials have many potential applications, such as imaging, sensing, and diagnosis [16].

Currently, there is a need of developing simple methods for obtaining NPs, accessible and scalable, with low costs. Plant extracts meet these goals, being easy to grow at a large scale, renewable, and environmentally friendly. In the synthesis process of metallic NPs, phytochemicals play two roles: (1) as a reducing agent and (2) as a stabilising agent for NPs [17,18]. Plant-mediated BNPs are more stable and varied in shape and size. Thus, the reducing agent in the plant extract (flavonoids, terpenoids, phenolic acid) is essential for the synthesis of BNPs [19]. Flavonoids are the most famous polyphenols present in many fruits and vegetables in the form of flavones, isoflavones, flavonols, and anthocyanins. Phenolic acids, such as salicylic acid, syringic acid, and gallic acid, are common in many plant species, and lignans are abundant in cabbage, broccoli, carrots, or cereals [20,21,22,23,24].

Various applications of plant-mediated BNPs are due to the synergistic effect of metal ions. Therefore, systems such as Ag–Au BNPs, Au–Pt BNPs, Ag–Pd BNPs, Ag–Cu BNPs, Ag–Ne BNPs, and Fe–Ag BNPs obtained with various plant extracts with potential antibacterial, antifungal, antidiabetic, and anticancer efficacy have recently been studied [25,26,27,28].

This review focuses on the synthesis and characterisation of plant-mediated BNPs and their pharmacological potential. It is also discusses the advantages of bimetallic nanoparticles over monometallic ones.

## 2. Research Methodology

The literature data were collected via PubMed, Clarivate Analytics, Science Direct, and Google Academic (2011–2021). The search terms were: “bark extracts”, “bimetallic nanoparticles”, “silver nanoparticles”, “gold nanoparticles”, “platinum nanoparticles”, “palladium nanoparticles”, “phytochemicals”, “green synthesis”, “anticancer”, “antiproliferative effect”, “antioxidant activity”, “antibacterial activity”, and “antidiabetic activity”. All of the search terms listed above were used in different combinations.

BioRender was used for drawing the manuscript figures (https://app.biorender.com, accessed on 27 September 2021) [29].

## 3. Synthesis and Characterisation of BNPs

Biosynthesis of BNPs using natural extracts has many advantages, being preferred by researchers to the detriment of chemical synthesis. The natural way of obtaining is simple, economical, and environmentally safe, with high reproducibility, even at the industrial level [10,30]. In chemical synthesis, the products obtained are unstable, requiring stabilising and protective agents, which are generally toxic and expensive. In contrast, plant extracts contain such substances, which act as reducing agents (flavonoids, terpenoids, tannins, alkaloids) or stabilising agents (carbonyl, carboxyl, and amine groups) of BNPs. In addition, plant extracts do not contain toxic by-products [31].

BNPs containing silver, gold, platinum, and palladium (noble transition metals) are of great interest for biomedical applications because they provide safety, biological stability, and low toxicity. However, two metal sources are needed to synthesise BNPs (e.g., Pd^2+^ and Au^3+^) [30]. Moreover, the reaction time, the pH of the reaction solution, the temperature, and the concentration of the metal salt depends on plant extract composition and considerably affect the size, morphology, and quality of the NPs [19].

In general, the NPs synthesis method includes two preparation processes: from top to bottom (by disassembling larger objects) [25] and from the bottom to up (by reducing metal cations) [26]. The top-down approach begins with the bulk material and fragmentation by external mechanical forces in the presence or absence of catalysts. This method is performed by various techniques, such as evaporation-condensation, laser ablation, or other physical methods. Although this is a faster method, there is no control over the shape and size of BNPs [32]. However, these procedures have several disadvantages, as they require the use of toxic chemicals, produce harmful by-products, and require high energy consumption [33]. In addition, these processes are quite difficult to extend if industrial-scale manufacturing is required [32,34]. The bottom-up approach begins at the atomic and molecular level, which is assembled at the nanoscale level. This method is performed by various biological and chemical methods. In this method, the size and shape can be controlled by adjusting the synthesis parameters. This is a slower method [27,28,35]. Previous studies have shown that different biological pathways can be used to make nanoparticles using plants, bacteria, fungi, algae, and yeast, as they contain metabolites that can reduce metal salts and formulate nanoparticles [36]. In addition, these substances do not only act as a reducing agent, as they are simultaneously involved in the stabilisation of nanostructures. Another advantage of the biological synthesis route is the possibility of production on an industrial scale. To date, there is no known operation for the industrial manufacture of nanoparticles by these techniques. However, this would be beneficial as the main raw material is renewable. The process would require minimal energy, resulting in low operating costs and almost negligible toxicity of the waste [37,38,39].

The general protocol for plant-mediated BNPs synthesis (bottom-up) is based on:

(a) A sequential reduction can perform the formation of the core-shell NPs. For example, metal salt can be reduced by the plant extract, followed by adding a second metal ion, producing a core-shell BNP. The formation of BNPs can be easily monitored by spectral changes in the UV-visible region due to the formation of the plasmonic absorption band [40,41]. Core-shell NPs are the most common type of composite NPs due to the integrated functionality of the core and coating. The physical and chemical properties of nanostructured composites, such as optical, magnetic behaviour or chemical reactivity, can be adjusted by changing the synthesis conditions in correlation with the thermodynamic parameters [42].
(b) The inorganic salts (s) (e.g., silver nitrate + gold chloride acid) are dissolved in water, followed by the addition of a plant extract, stirring continuously at room temperature. Therefore, if both metal precursors are present simultaneously, then their simultaneous reduction can lead to the formation of alloy nanoparticles (e.g., Au–Ag BNPs). For example, Au–Ag alloy NPs have been shown to have a synergistic effect on carbon monoxide (CO) oxidation [40,41].

The characterisation of nanoparticles is significant for evaluation, synthesis control, and future applications. Thus, the most used characterisation techniques are UV–VIS (ultraviolet-visible spectroscopy), FT-IR (Fourier transform infrared spectroscopy), UHPLC (ultra-high-performance liquid chromatography), TEM (transmission electron microscopy), SEM (scanning electron microscopy), EDX (energy-dispersive spectroscopy), DLS (dynamic light scattering), zeta potential, and XRD (powder X-ray diffraction) [43,44]. UV–VIS is based on the detection of surface plasmon resonance (SPR), which can be described as an interaction between light and matter [45]. FT-IR is applied in elucidating the structure and identifying a chemical compound. The main objective of IR spectroscopy is to determine the functional groups of a sample by absorbing IR radiation at characteristic frequencies [46]. TEM and SEM characterise the size and morphological forms of the synthesised BNPs [14]. EDX studies the purity of synthesised BNPs [47]. DLS identifies BNPs agglomerations and determines the distribution and size of BNPs in solution [13]. The zeta potential is based on the long-term stability of the solution [48]. XRD identifies the crystallinity of BNPs [49].

Elemike et al. reported the biogenic synthesis of Au–Ag BNPs using *Stigmaphyllon ovatum* extract. The synthesis of BNPs was monitored, and the absorption was observed around 534 nm after 30 min, and changed at 542 nm after 45 min; changing colour to red was confirmed by the presence of the band plasma resonance (SPR) of Au–Ag BNPs obtained in UV–VIS spectra [46]. In a similar approach, Au–Ag BNPs (alloy structure) were obtained using leaf extract with golden stem (*Solidago canadensis*). BNPs were monitored and characterised using absorbance peaks (UV–VIS spectrophotometer—absorption at 530 nm), particle size, and diffraction patterns, and synthesised NPs morphologies were characterised by SEM and TEM. TEM images showed that Au–Ag BNPs have spherical shapes, but due to occasional aggregations, some rod-like triangular shapes were found in Au–Ag BNPs [14]. Sivamaruthi et al. reported Pd–Ag BNPs biosynthesis from an aqueous fruit extract of *Terminalia chebula*. The used extract is rich in antioxidant agents such as polyphenols. XRD analysis and DLS analysis confirmed the formation of the cubic crystal structure of Pd–Ag BNPs with average dimensions of 20 nm. Uniform spherical NPs were observed in the SEM and TEM analysis. BNPs do not show any significant SPR peak similar to PdNPs [47]. Therefore, non-plasmonic NPs also depend on size, concentration, and optical properties. However, their spectrum is not as sensitive to dispersion properties as plasmonic NPs [48]. Ag–Pt BNPs were also characterised using different methods: UV–VIS, FTIR, electron microscopy, and X-ray diffraction analysis [49]. Another study also reported the rapid and compelling synthesis of Au–Ag BNPs using aqueous *Plumbago zeylanica* root extract. FTIR confirmed bioreduction; EDS and XRD confirmed the crystalline nature and purity of NPs, respectively; and TEM and DLS micrographs confirmed that AgNPs are spherical (60 nm), AuNPs are anisotropic in nature, with spheres, triangles, and hexagons (approximately 20–30 nm), and a unique feature of Au–Ag BNPs is their shape: polygonal NPs with the blunt hexagonal end (around 90 nm). All these various properties are due to the rich extract in flavonoids, sugars, organic acids, phenols, starch, and citric acid [50].

Other BNPs mediated by plant extracts are Au–Ag BNPs, Ag–Pt BNPs, Ag–Pd BNPs, Au–Pt BNPs, Au–Pd BNPs, Pt–Pd BNPs, etc. Regarding the combination of silver and gold NPs, the studies showed about 3–80 nm dimensions and hexagonal, triangular, and spherical shapes. Moreover, regarding the combination of silver and palladium NPs, the studies showed approximately 7–70 nm dimensions and spherical and cubic forms. More details on the types, sizes, and shapes of NPs are disclosed in Table 1.

## 4. Factors Influencing BNPs Biosynthesis

Factors affecting the biosynthesis of BNPs are pH, temperature, reaction time, and metal ion concentration. The size and shape of the NPs depend on chemical and physical factors. Optimal metal ion concentration, temperature, and pH of the reaction mixture play critical roles in nanoparticle synthesis [48,76].

Therefore, it is possible to resort to the centrifugation of the obtained NPs solutions to obtain pure BNPs and avoid the interaction of several compounds (e.g., organic dyes) used in the analysis methods. Furthermore, centrifugation at 10,000–15,000 rpm for 15–20 min leads to the isolation of the NPs and their redispersion in distilled water [77,78,79].

### 4.1. The pH of the Solution

The pH value is an essential experimental parameter in the growth dynamics of NPs. Therefore, pH can influence most of the equilibria involved in the process [70]. For example, at a certain pH, the surface charge can be reduced to zero; this is called the isoelectric point [48]. Several studies have confirmed that pH has an important role in controlling the size and formation of NPs obtained [1,19]. Ganaie et al. reported that there was an increase in optical density with increasing the pH. At pH 10, the Ag–Au BNPs synthesis was completed within minutes of the start of the synthesis. When Au–Ag BNPs were obtained by the co-reduction method at pH 4–6, the reactants had two peaks (each in regions of 500–550 and 400–420 nm), and at pH 7, the reactants had a single peak (approximately 490 nm) [80]. Akilandaeaswari et al. reported the effect of pH (5, 6, 7, 8, 9) on Au–Ag BNPs synthesised from Lawsonia inermis extract. The recommended optimal condition was at pH 7. Thus, the obtained BNPs showed an excellent catalytic activity for the reduction/degradation of 4-nitrophenol to 4-aminophenol in the presence of NaBH_4_ [81].

### 4.2. Reaction Temperature

Temperature is a physical parameter; it plays an essential role in the spatial and dimensional distribution of particles, especially in the case of Au–Ag BNPs, Ag–Pt BNPs, and Au–Pd BNPs [51,82]. The experimental determinations based on the approached procedure aim at optimising the experimental parameters of the studied biosynthesis process, taking into account the changes in the UV–VIS spectrum. Therefore, increasing the reaction temperature increases and the rate of reduction of metal ions [83], so at 70 °C, smaller nanoparticles are formed with the help of *Canna indica* extracts [84]. Olawale et al. reported that Ag_2_Se BNPs synthesis was observed at high temperatures (110 °C) along with a large volume of extract (500–1700 μL). Therefore, the analysis showed that the increase in the temperature leads to an increase in the reaction rate. Moreover, the increase in the amount of extract could improve the yield by providing more reducing agents necessary for the complete reduction of metal ions [85]. Moreover, in the case of extracts from leaves of *Ocimum tenuiflorum*, the rate of synthesis of nanoparticles increased after one minute to 79% under conditions of a temperature of 95 °C and after 5 min to 100%. The high temperature favours the fast conversion of the metallic solution into nanoparticles, with the synthesis being longer at room temperature [51,86].

The temperature increases the kinetic energy of the reactants. The increase in temperature catalyses the formation of nanoparticles by increasing the formation of nucleation centres due to the rapid reduction of metal cations [87]. In addition, the high temperature leads to the formation of stable and smaller nanoparticles. It has also been reported that the absorbance of nanoparticles increases with temperature, indicating a high concentration of synthesised nanoparticles. However, the temperature must be maintained in the range of 30–100 °C, as phytochemicals decompose at higher temperatures, interfering with the critical reduction process [83,84].

### 4.3. Reaction Time

Biosynthesis time is an important parameter to consider because the reduction reaction, regardless of the extract used, cannot take place instantly [1]. It has been observed that colour transformation is dependent on the time and temperature at which BNPs biosynthesis takes place [88]. Elemike et al. [46] performed the green synthesis of Ag NPs, Au NPs, and Ag–Au BNPs using *Stigmaphyllon ovatum* leaf extract. Au–Ag BNPs were synthesised by adding 250 mL of 1 mM HAuCl_4_ + 250 mL of 1 mM AgNO_3_ in 100 mL of aqueous extract, and the solution was heated by stirring at 80–85 °C for 1 h. The reaction process was monitored using UV–VIS spectroscopy for different time intervals. Ag NPs formed at 90 min, with absorption taking place at around 420 nm, while Au NPs formed at 60 min and absorption took place at around 550 nm. In contrast, UV–VIS analysis of Au–Ag BNPs revealed earlier formation of plasmonic bands after 15 min of reaction; the intensity of the band increased over time. The absorption was observed after 30 min, around 534 nm, which changed to red after 45 min at 542 nm. Thus, increasing the reaction time will increase the ion reduction rate until the reaction reaches the end. The formation of two bands in the bimetallic nanoparticles reflects the core shell, but in this case, a band from the Au region showing more Au than Ag was reduced, and there was possible nano alloy formation [46]. Akilandaeaswari et al. reported that Au–Ag BNPs were obtained continuously by changing the synthesis time from 2 to 40 min; after 10 min, the colour of the reaction mixture changed from pale yellow to dark purple [81]. The UV–VIS absorption spectra of Au–Ag BNPs synthesised at pH 4 by the simultaneous addition of Au (I) and Ag (I) (15 mg/L) each and the root extract concentration of 1200 mg/L showed an increase marked optical density over time to peak, indicating the highest BNP concentration achieved in that synthesis. For three months, the absorption remained unchanged. At pH 10, the formation of BNPs was complete within minutes of the start of the synthesis; therefore, the optical density reached its peak after one hour [80]. Gopinath et al. [15] performed the green synthesis of Ag NPs, Au NPs, and Au–Ag BNPs using *Gloriosa superba* leaf extract. Nanocomposites were synthesised by the addition of 5 mL of *Gloriosa superba* leaf extract, which was mixed with 100 mL of each of the following solutions: 1 mM silver nitrate (pH 4.35), 1 mM chloroauric acid (pH 2.26), and silver/acid nitrate chloroauric acid (1:1) (pH 5.2) at room temperature. The investigation of the samples was performed by UV–VIS spectrum between 200 and 800 nm and at different time intervals, such as 0, 1, 2, 3, 4, 5, and 10 min. After 10 min, reduction in Ag NPs, Au NPs, and Ag–Au BNPs was visually detected. The initially uncoloured solution was rapidly coloured brown (for Ag NPs), yellow to dark red (for Au NPs), and yellowish white to reddish brown (for Ag–Au BNPs). The spectra recorded for the leaf extract did not show any absorption peak between the region of 200 to 850 nm; instead, Ag NPs showed an absorption peak at 425 nm, Au NPs showed an absorption peak at 538 nm, and Au–Ag BNPs showed an absorption peak at 559 nm [15].

### 4.4. Metal Ion Concentration

The initial concentration of metal ions in the reaction mixture is another crucial factor [80]. In a study by Mittal et al. [89], Ag–Se BNPs were synthesised using different quercetin and gallic acid concentrations as a bioreduction and coating agent. The research used different dilutions of quercetin and gallic acid (10–1000 μL) from the stock solution (10 mM), which were prepared in water and further diluted to 100 mL with deionised water to make the reducing solution of different concentrations. The resulting solutions were reacted with a mixture of silver and selenium salt (0.5–5 mM) by incubation at 35 °C (200 rpm) under dark conditions. Thus, a mixture of quercetin and gallic acid (50 μM, each) gave a maximum yield of bimetallic nanoparticles. The effect of AgNO_3_ and Na_2_SeO_4_ concentrations were studied from 0.5 to 5 mM using 50 μM quercetin and gallic acid each. An increase in the yield of Ag–Se NPs was observed when the metal salt concentration increased from 0.5 mM to 1 mM. The nanostructures were characterised by using the UV–VIS spectrum, which displayed a surface plasmon absorption band at 450 nm. A general trend was found in which the SPR tip shifted to the region with a longer wavelength and became narrower as the concentration increased [89]. Thus, a concentration of 1 mM leads to a rapid metal reduction due to the availability of functional groups in the extract compounds [67,68]. Instead, a 5–10 mM concentration increases the size and aggregation of NPs due to the competition between metal ions and functional groups. Higher concentrations of metallic solutions (e.g., AgNO_3_, Cu(CH_3_COO)_2_, HAuCl_4_, Na_2_PdCl_4_) lead to the formation of larger NPs [66,73].

### 4.5. Plant Extract/Biomass Dosage

The plant extract used is also dependent on certain factors: (1) the plant species, (2) the solvent used for extraction (the most used is water), and (3) the temperature of extraction [77,81]. Each plant has particularities in the phytochemical composition that will influence the obtaining of different NPs sizes. The synthesis of Ag–Au BNPs using *Gloriosa superba* leaves extract has been completed after 10 min at room temperature by visual detection. The initial white-yellow solution was rapidly stained to reddish-brown, indicating the rapid formation of Ag–Au BNPs [15]. Moreover, the presence of reducing agents in larger quantities in the *Lawsonia inermis* seed extract led to an increase in the intensity of the absorbents, visualising that the nanoparticles obtained are smaller and have a spherical shape [81]. After analysis with a UV–VIS spectrophotometer, it was observed that the intensity of the absorbents increased with the increase of the concentration of pomegranate extracts *(Punica granatum)* [90].

## 5. BNPs vs. Monometallic NPs

BNPs composed of two different metals have attracted more interest than monometallic NPs, both scientifically and technologically [74]. BNPs synthesised with different biological methods (plant extracts, bacteria, viruses, yeasts, fungi) have more potential than monometallic NPs synthesised with biological methods, due to an additional degree of freedom [41]. All these biological methods of BNPs synthesis have different advantages and disadvantages. Compared to microbial synthesis, plants can be conveniently used for the production of NPs [91]. The use of plant extracts can easily extend the synthesis of NPs. In addition, plant extracts can reduce metal ions faster than microbes and produce stable metal NPs [92]. In plant extracts, there are many active compounds (terpenoids, alkaloids, phenols, tannins, and vitamins) that act as coating and reducing agents [93]. The active compounds of bacteria and fungi are intracellular enzymes; the sugar molecules, canonical membrane proteins, enzymes dependent on nicotinamide adenine dinucleotide (NADH) and nicotinamide adenine dinucleotide phosphate (NADPH), act as reducing agents. A disadvantage of plant extracts and fungi is that the bacteria are relatively inexpensive to grow and have a high growth rate [94].

The biological activity of NPs depends on their size and shape. As shown in Table 2, the size of the NPs varies depending on the type of salt used. Morphological variations of NPs are closely related to the type of metal, the salt concentration, the reaction mixture’s physical conditions, and the used plant extract for the biosynthesis of the NPs [95].

### 5.1. Advantages of BNPs vs. MNPs Synthesised from Green Extracts

BNPs synthesised from natural extracts have some advantages, such as the temperature of synthesis (room temperature) and the lack of toxic solvents. It is an ecological technique and cost-effective [96]. BNPs have characteristic mixing patterns and geometric architecture that improves their functionality. Thus, homogeneous NPs are produced [12]. Natural extracts are used as a reducing and stabilising agent for BNPs [97]. BNPs have a substantial impact on human health (applications in medicine and pharmacy) due to the proven biological activities (catalytic, antioxidant, antibacterial, antidiabetic, antitumor, hepatoprotective, and regenerative) and the synergistic effect between the two metals [77]. Metallic NPs can remove free radicals, while BNPs synthesised at low reactant concentrations are more active [98]. BNPs containing silver, gold, platinum, and palladium (noble transition metals) are of great interest for biomedical applications because they provide safety and stability in the biological environment, as well as having low toxicity [99].

### 5.2. Disadvantages of BNPs vs. MNPs Synthesised from Green Extracts

Heating conditions, such as rising temperatures, are the required cost of NP production [12]. The mechanism of synthesis of BNPs must also be elucidated, and the reproducibility of biogenic processes must be improved [96,100,101]. Moreover, high concentrations of both salts (1 M concentration each) led to an increase in the size of BNPs [102]. NPs have a biological potential that deserves to be developed, requiring more studies on the mechanism of action in living systems. Consequently, it is necessary to develop new synthesis methods and to optimise the properties of NPs with noble metals [103]. Furthermore, studies are needed to establish the link between BNPs solubility and bioavailability in organisms. Dissolution is an essential process for the bioavailability of BNPs. This process is influenced by the BNPs size, pH, and the presence of ligands in the reaction medium [10,100,101]. Moreover, exposure routes and transformation processes make it difficult to predict the bioavailability of NPs. An important aspect is that BNPs studies should contain more detailed information about biochemical and chemical conversion in the reaction medium.

## 6. Applications of BNPs Mediated by Plant Extracts

**Figure 1:**
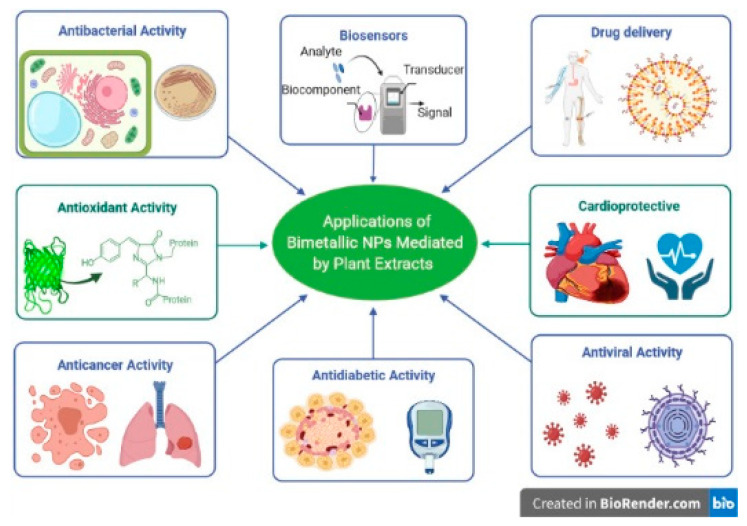
Applications of bimetallic nanoparticles (BNPs) mediated by plant extracts (created with BioRender.com (accessed on 27 September 2021)) [29].

The applicability of BNPs is more compared to MNPs due to the enhancement of properties due to the small size effect, quantum size effect, surface effect, and quantum tunnelling effect [15]. BNPs have been used in household use, in the cosmetics industry, and in food storage, among others. An objective of this review is to emphasise the applications of BNPs in different biological fields and biomedical applications (antibacterial, antifungal, antiviral, anti-inflammatory, anticancer activity) [77]. A schematic diagram representing various applications of BNPs is provided in Figure 1.

### 6.1. Antioxidant Activity

Phytochemicals present in plants are well studied, especially polyphenols (such as flavonoids and phenolic acids) [52]. Flavonoids protect the body from free radicals, help strengthen the immune system, and reduce inflammation. They are responsible for the antioxidant activities of plants. These natural compounds contain one or more hydroxyl groups related to the carbon atoms of the aromatic ring [104,105]. Many phytochemicals are placed directly under the bark or in the outer leaves. They are specific to every plant, even every plant cell. Some authors also believe that phytochemicals together with NP of noble metals reduce the risk of lung, breast, or colon cancer [52,106,107]. This is due to the antioxidants that fight the devastating effects that free radicals have [105,107]. Two mechanisms for antioxidant activity can be classified as (1) a method of hydrogen atom transfer (HAT) that donates a hydrogen ion from a stable molecule, thus allowing the antioxidant to eliminate ROS, and (2) a single electron transfer (SET), which depends on the antioxidant’s potential to reduce certain molecules and compounds by transferring an electron [71,104,106]. Therefore, it is concluded that the antioxidants present in plants are responsible for the green synthesis of metal nanoparticles or metal oxides due to their ability to reduce or chelate metal ions and to act as stabilisers of the NPs produced [108].

NPs of noble metals (Au, Ag, Pt, Pd) were synergistically enhanced by becoming BNPs (Au–Ag BNPs, Au–Pt BNPs, Pt–Pd BNPs, etc.) with antioxidant action. The antioxidant activity of certain compounds can be demonstrated in several ways. One of the well-known methods is based on the discolouration of the stable radical DPPH (2,2-diphenyl-1-picrylhydrazyl).

In recent studies, it has been shown that antioxidant activity is two times higher for BNPs compared to monometallic NPs [49,109,110]. Adebaya et al. showed that Au–Ag BNPs obtained from the aqueous extract of *American persea* fruit peel showed antioxidant activity. [111]. Ag–Pt BNPs synthesised from *Crocus sativus* have higher antioxidant properties compared to Ag NPs and Pt NPs, respectively. Inhibition of DPPH radicals of biosynthesised Ag–Pt BNPs was greater than the ABTS radical. This aspect is due to the H^+^, and it can break free radical chains [49]. Pt–Pd BNPs synthesised from *Peganum harmala* showed a higher antioxidant activity compared to monometallic NPs. Thus, the antioxidant activity for Pt–Pd BNPs was 843.0 ± 60 μM TE/mg NPs, for Pt NPs was 277.3 ± 13.5 μM TE/mg NPs, and for Pd NPs was 167.6 μM 4.8 μM TE/mg NPs. In addition to its antioxidant activity, it also has anticancer activity against lung cancer (A549) and breast cancer (MCF-5) [112]. Another study showed that Ag–Ni BNPs synthesised from *Salvadora persica* showed antioxidant activity. It was demonstrated by three methods: (1) the mechanism of DPPH free radical scavenging, (2) the phosphomolybdenum complex method, and (3) the determination of phenolic content [113]. The antioxidant activity of noble metal NP synthesised from plant extracts is associated with a reduced risk of diseases, such as cancer and cardiovascular disease [114].

### 6.2. Antibacterial Activity

Noble metal NPs are of great interest for biomedical applications [55]. Some researchers have reported that BNPs show higher antibacterial activity for Gram-positive and Gram-negative bacteria compared to MNPs [72,89,115]. Sivamaruthi et al. reported that Ag–Pd BNPs synthesised from the fruit extract of *Terminalia chebula* exhibits amazing antibacterial activity aginst MRSA, methicillin-sensitive *Staphylococcus aureus* (MSSA), and *Pseudomonas aeruginosa*. The inhibition areas for these bacteria were 12, 14, and 16 mm, respectively, compared to monometallic NPs that showed no significant antimicrobial activity against these tested microbial strains [47]. The antibacterial activity of bimetallic nanoparticles synthesised from green extracts appears to be more attractive than their monometallic counterparts due to the synergistic effects between the two different metals [113]. BNPs showed superior performance compared to conventional antibiotic treatments against several Gram-positive and Gram-negative bacteria, viruses by their synergistic anti-microbial efficiency [108]. Many studies show that BNPs have antibacterial activity against Gram-positive bacteria: *Streptococcus pneumonia*, *Clostridium tetani*, *Clostridium difficile*, *Staphylococcus aureus*, and *Bacillus anthracis*. Moreover, BNPs have antibacterial activity against Gram-negative bacteria: *Escherichia coli, Pseudomonas aeruginosa, Neisseria gonorrhoeae, Vibrio cholerae*, *Chlamydia trachomatis*, and *Yersinia pestis* [15,116,117]. Sundarrajan et al. demonstrated that Ag–Au BNPs synthesised from the fruit extract of *Artocarpus heterophyllus* showed lower antibacterial activity against Gram-positive bacteria and higher antibacterial activity against Gram-negative bacteria [55]. BNPs presented an excellent antibacterial activity compared to commonly used antibiotics. Therefore, pathogens cannot develop resistance to them because they suppress the generation of biofilms and accelerate other related processes [50,118].

**Figure 2:**
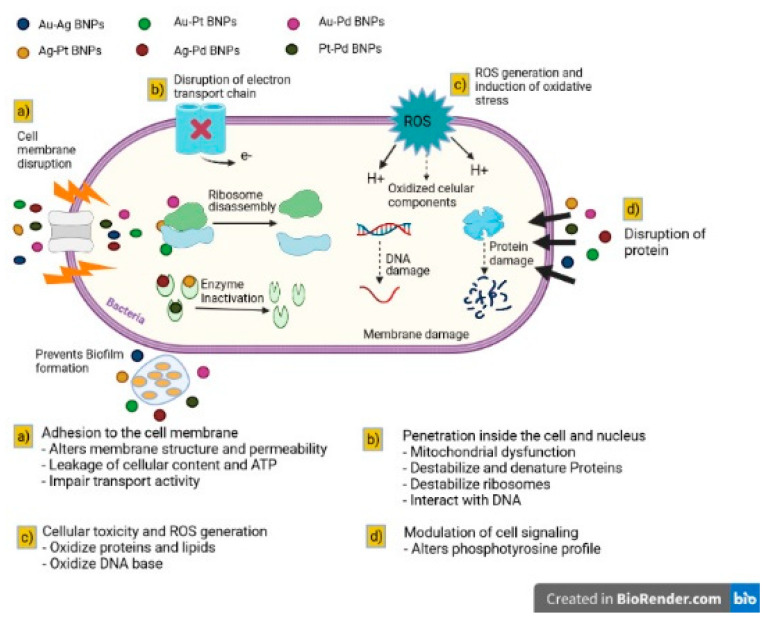
General mechanisms for the antimicrobial mode of action of bimetallic nanoparticles (BNPs) (created with BioRender.com (accessed on 27 September 2021)) [29].

As shown in Figure 2, BNPs can interfere with bacterial growth through various mechanisms: (1) adhesion to the cell membrane: changes the structure of the membrane and permeability, cell and ATP secretions, and transport activity with deficiencies; (2) penetration inside the cell and nucleus: mitochondrial dysfunction, destabilises and denatures proteins, destabilises ribosomes and interacts with DNA; (3) cellular toxicity and ROS generation: oxidises proteins, lipids, and the bases of DNA; (4) modulation of cellular signalling: modifies the phosphotyrosine profile [103,119,120].

Ag–Au BNPs synthesised from *Gracilaria* sp. showed antibacterial activity against *Klebsiella pneumoniae* and *Staphylococcus aureus* [121]. Au–Pt BNPs obtained through the mediation of natural extracts showed antibacterial activity against *Candida albicans, Pseudomonas aeruginosa*, and *Staphylococcus aureus* [122]. Cu–Ag BNPs obtained from the *African kigelia* fruit extract demonstrated antibacterial activity [72]. Pd–Ag BNPs obtained from the fruit extract of *Terminalia chebula* proved antibacterial activity against MRSA and *Pseudomonas aeruginosa*. Moreover, these Pd–Ag BPNs demonstrated anticancer activity; in vitro toxicological studies showed that Pd–Ag BNPs did not present cytotoxic and hemolytic effects up to a maximum dose of 200 μg/mL, ensuring the biocompatibility of NPs [47]. Furthermore, Au–Ag BNPs synthesised from *Ocimum basilicum* leaf extract showed antibacterial activity against *Staphylococcus aureus, Pseudomonas aeruginosa*, *Escherichia coli*, and *Bacillus subtilis*; in addition, Au–Ag BNPs presented an antidiabetic activity [123].

Merugu et al. demonstrated the antioxidant, antibacterial, and antitumor activity of Ag–Cu BNPs and Cu–Zn BNPs using toddy palm. Antibacterial activity was demonstrated against *Alcaligenes faecalis, Staphylococcus aureus*, *Citrobacter freundii*, *Klebsiella pneumoniae*, and *Clostridium perfringens* [108].

### 6.3. Anticancer Activity

**Figure 3:**
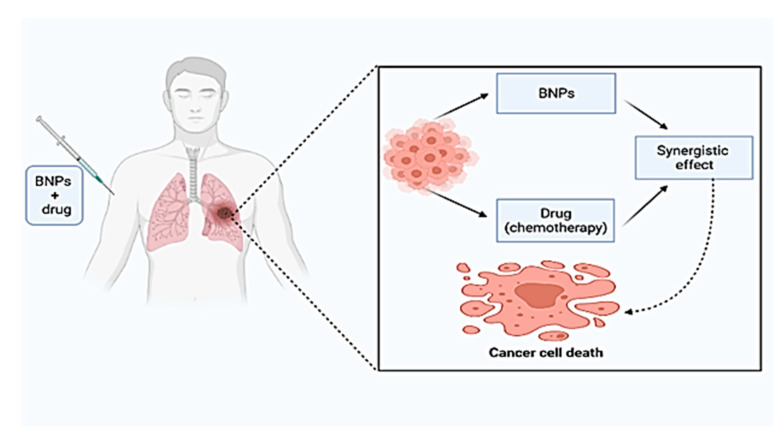
Treatment of tumour cells by bimetallic nanoparticles (BNPs).

Cancer is a term used to define malignancies in which abnormal cells multiply uncontrollably and can invade the surrounding healthy tissues. Abnormal cells come from any tissue in the human body and can occur anywhere in the body [103]. The BNPs act as chemotherapeutic agents in treating tumour cells and show a synergistic effect with drug chemotherapy (Figure 3).

Many studies show that BNPs have anticancer activity [45,54,60,71,124]. It was reported that BNPs have a cytotoxic effect against various types of cancer cells, such as the breast cancer cell line [46,89]. The anticancer activity is due to the synergistic effect between the two metals used compared to the monometallic NPs [103]. Some researchers have suggested that cancer cells are more susceptible to electron transfer between BNPs that release reactive oxygen species (ROS) and thus destroy cancer cells such as MCF-7 (breast cancer cell line), HeLa (breast cancer cells), Jurkat (human T lymphocyte cells), HT-29 (human colon cancer cell line), HEK 293 (human embryonic renal cells), and T24 (human bladder cancer cells) [60,125]. Given the size of the metallic NPs and the concentration of the sample, researchers observed that BNPs could cause damage to a cancer cell. Thus BNPs are dose- and size-dependent [103,112]. For example, Au–Pt–ZnO TNPs synthesised from Arctium lappa extract showed anticancer activity; this activity was demonstrated by the MTT test when the concentration of the tested NPs was 10 mol [126].

For breast cancer cells, BNPs have a high affinity and specificity [86]. Pt–Pd BNPs showed anticancer activity against lung cancer (A549) and breast adenocarcinoma cells (MCF-5) with an IC_50_ of 8.8 g/mL and 3.6 g/mL, respectively [112]. Ghosh et al. reported that Pt–Pd BNPs (74.25%) showed higher anticancer activity against HeLa cells compared to Pt NPs (12.6%) and Pd NPs (33.15%). Therefore, the total number of cells subjected to cell death was much higher in Pt–Pd BNPs [71]. Sharma et al. demonstrated that Ag–Cu BNPs exhibit strong anticancer activity against breast cancer (MDA-MB-231). IC_50_ values calculated after 24 and 48 h of incubation were 19.27 μg/mL and 6.99 μg/mL, respectively [73]. Ag–Pt BNPs were synthesised using ethanolic plant extract of *Vernonia mespilifolia*; they showed a selective cytotoxic potency against the MCF-7 breast cancer cell line compared to the normal HEK 293 cell line. Lethal concentration calculated for Ag–Pt BNPs on HEK 293 and MCF-7 cells was 60 μg/mL and 10.2 μg/mL, respectively [60]. Au–Ag BNPs have cytotoxic effects for different human cancer cell lines (HepG2, MDA-MB-231, MCF-7) [127]. Another study also showed that Au–Ag BNPs synthesised from *Desmodium gangeticum* showed excellent results against prostate cancer (DU 145) and cervical cancer (HeLa) using the MTT reduction test [128]. Mittal et al. demonstrated the efficacy on Dalton lymphoma (DL) cells of BNPs (Ag–Se BNPs) synthesis mediated by quercetin (flavonoid) and gallic acid (polyphenol) [89]. Merugu et al. demonstrated the antitumor activity of Ag–Cu BNPs and Cu–Zn BNPs using toddy palm; the cytotoxicity study was performed against nasopharyngeal cancer (KB) cells and Ehrlich ascites carcinoma cell lines [108].

In conclusion, BNPs have an affinity for tumour cells due to their characteristic acidic pH. For this reason, BNPs have the advantage of targeting tumour cells and minimising side effects on healthy cells [129,130].

### 6.4. Antidiabetic Activity

Diabetes is a metabolic disease that causes excess glucose in the blood (hyperglycemia), being represented by the deficiency of insulin secretion or insulin resistance. Diabetes is the third leading cause of death worldwide after cardiovascular disease and cancer [131,132]. α-Glucosidase and α-amylase (porcine) enzymes are considered the key antidiabetic enzymes that metabolise carbohydrates. The enzymatic activity of human pancreatic α-amylase and α-glucosidase in the small intestine correlates with postprandial increased glucose levels. Therefore, amylase and glucosidase inhibitors prevent the breakdown of carbohydrates into monosaccharides, the main reason for the increased level of glucose in the blood [133]. Alpha-glucosidase is an enzyme that participates in the process of digesting carbohydrates. It mediates the cleavage of polysaccharides and disaccharides to glucose. Thus by inhibiting α-glucosidase, it delays the digestion and absorption of carbohydrates [134]. Synthesised BNPs, such as Au–Ag BNPs and Pt–Pd BNPs, using various plant resources, show antidiabetic activity. Those plant resources contain secondary metabolites such as saponins, flavonoids, steroids, alkaloids, and tannins that play an essential role in controlling diabetes [132]. In several studies (in vivo, in vitro), BNPs have been considered α-amylase inhibitors [135,136,137].

For example, biogenic Au–Ag BNPs synthesised from the aqueous extract of *Ocimum basilicum* leaves and flowers showed significant in vitro antidiabetic efficacy; 69.97 ± 3.42% inhibition was observed against the α-amylase enzyme, and 85.77 ± 5.82% inhibition was observed against α-glucosidase enzyme [123]. Another study also showed that Au–Ag BNPs synthesised from aqueous *Trigonella* seed extract have antidiabetic activity [138].

### 6.5. Other Activities

BNPs also show substantial antifungal activity against *Candida albicans* [30,139]. Ag–Cu BNPs show antiviral activity on strains HHV-1, human alphaherpesvirus 1, and HHV-2, human alphaherpesvirus 2 [140]. Catalytic activity is dependent on the reduction of 4-nitrophenol to 4-aminophenol in the presence of NaBH_4_. Au–Ag alloy BNPs showed the highest catalytic activity compared to all other NPs [58]. In addition to antibacterial activity, Au–Ag BNPs (synthesised from *Asparagus racemosus* root extract) also showed immunomodulatory activity. Immunomodulatory activity is performed by measuring the level of cytokines in macrophages using the solid-phase sandwich ELISA technique [53].

A recent study showed that Au–Pt BNPs synthesised from *Citrus limon* leaf extract exhibits larvicidal activity at the selected concentration [67]. Ag–Co BNPs synthesised from *Borassus flabellifer* also exhibit larvicidal activity [141]. Other applications of metallic NPs can be found in Table 3.

### 6.6. Biocompatibility of BNPs

The results about the biocompatibility of BNPs are necessary before their use in the biomedicine field. In general, several phytomolecules (phenols, tannins, flavonoids, terpenoids, vitamins) are responsible for the properties of BNPs. These biomolecules are the key factor responsible for the mechanism of BNP action [151]. To date, various methods (qualitative and quantitative) have been used to assess the biocompatibility of BNPs. The BNP type (containing silver, gold, platinum, and palladium), as well as the size, shape, concentration, and surface properties of BNPs, have an important role in their biocompatibility [10]. However, information about the biocompatibility of BNPs requires future research.

## 7. Conclusions and Future Perspectives

This review was written to gain insight into the synthesis and characterisation of BNPs, as well as their possible applications. Many researchers prefer the biological method at the expense of the physical or chemical methods. Most bioactivity studies have focused on antioxidant, antimicrobial, and anticancer effects. Thus, plant extracts are an excellent source of phytoconstituents. Various functional groups found in plants are involved in the reduction, synthesis, and stabilisation of BNPs. BNPs containing silver, gold, platinum, and palladium (noble transition metals) are of great interest for biomedical applications because they provide safety, biological stability, and low toxicity.

The general conclusion of the scientists is that BNPs synthesised from natural extracts can be exploited for their antioxidant, antimicrobial, and anticancer potential activities. BNPs can be used in various research fields, such as chemotherapy, diagnostics, biomedical devices, drug delivery systems, and cosmetics, as well as in the chemical, energy, electronics, and space industries. These studies should continually develop newer techniques for reducing drug resistance microorganisms to antibiotics by using polyphenolic extracts. Therefore, more studies should focus on in vivo experiments. In these studies, issues such as excretion or non-targeted distribution of BNPs should be evaluated. In addition, more studies are needed to show the link between BNPs biosynthesis and bioactivity and to discuss their mechanism of action. Finally, although natural extracts are generally safe, more toxicological data are required.

## Figures and Tables

**Table 1:** Synthesis of bimetallic nanoparticles (BNPs) mediated by the extract plants.

Type of BNPs	Plant Species: Scientific Name (Family)—Common Name	Salt Concentration	Report Extract: Salt Solution	Synthesis Temperature	Time (min)	Size (nm)	Shape	Ref.
Au–Ag BNPs	*Solidago canadensis*: (Asteraceae)—Canada goldenrod	1 mM AgNO_3_ 1 mM HAuCl_4_·3H_2_O	1:5:5	70–80 °C with stirring	60	25.9	spherical	[51]
	*Geum urbanum* L.: (Rosaceae)—Wood avens	5 mM AgNO_3_ 5 mM HAuCl_4_	1:1:1	65 °C with stirring	720, 1080 and 1440	50	spherical, triangular, hexagonal	[52]
	*Asparagus racemosus*: (Asparagaceae)—Shatavari	1 mM HAuCl_4_ 1 mM AgNO_3_	1:5:5	irradiation with a frequency of 2.45 GHz and a power of 700 w	20	10–50	spherical	[53]
	*Moringa oleifera* Lam.: (Moringaceae)—Moringa	1 mM AgNO_3_ 1 mM HAuCl_4_	1:2:3	25 °C	25	109	spherical	[54]
	*Stigmaphyllon ovatum*: (Malpighiaceae)—Amazonvine	1 mM AgNO_3_ 1 mM HAuCl_4_	1:1:1	70–80 °C with stirring	60	15	spherical	[46]
	*Artocarpus heterophyllus*: (Moraceae)—Jack tree	3 mM AgNO_3_ 3 mM HAuCl_4_	1:1:1	solar heating	-	15	spherical	[55]
	*Commelina nudiflora* L.: (Commelinaceae)—Carolina dayflower	1 mM HAuCl_4_ 1 mM AgNO_3_	1:2:2	25 °C	120	20–80	spherical, rod, triangular	[56]
	*Madhuca longifolia*: (Sapotaceae)—Madkam	1 mM HAuCl_4_ 1 mM AgNO_3_	1:10:10	Ultrasonic liquid processors (20 kHz)	40	34–66	spherical	[57]
	*Gloriosa superba* L.: (Colchicaceae)—Flame lily	1 mM AgNO_3_ 1 mM HAuCl_4_·3H_2_O	1:10:10	25 °C	10	10	spherical	[15]
	*Pulicaria undulata*: (Asteraceae)	10 mM HAuCl_4_·3H_2_O 10 mM AgNO_3_	2:9:1	25 °C with stirring	120	5–12	spherical	[58]
	*Piper pedicellatum*: (Piperaceae)—Piper	1 mM AgNO_3_ 1 mM HAuCl_4_	1:15:15	30 °C with stirring	120	3.0–45.0	spherical	[59]
Ag–Pt BNPs	*Vernonia mespilifolia*: (Asteraceae)—Bitter tea	1 mM AgNO_3_ 1 mM K_2_PtCl_4_	1:5:5	85 °C with stirring	60	35.5 ± 0.8	spherical	[60]
	*Crocus sativus* L.: (Iridaceae)—Saffron crocus	1 mM AgNO_3_ 1 mM K_2_PtCl_4_	1:2:2	100 °C with stirring	60	36	spherical	[49]
Ag–Pd BNPs	*Terminalia chebula*: (Combretaceae)—Chebulic myrobalan	0.01 M PdCl_2_ 0.01 M AgNO_3_	-	25 °C	60	20–70	spherical	[47]
	*Catharanthus roseus*: (Apocynaceae)—Bright eyes	1 mM AgNO_3_ 1 mM PdCl_2_	1:4:4	25 °C	60	15–30	cubic	[61]
	*Cacumen platyclade*: (Cupressaceae)—Chinese Arborvitae Leaf and Twig	1 mM AgNO_3_ 1 mM PdCl_2_	1:4:4	25 °C	60	11.9 ± 0.8	spherical	[62]
			1:1:1			9.1 ± 0.7		
			1:1:3			7.4 ± 0.4		
	*Lithodora hispidula*: (Boraginaceae)	1 mM AgNO_3_ 1 mM K_2_PdCl_4_	1:2:2	25 °C with stirring	20	15.03–21.60	spherical	[63]
Au–Pd BNPs	*Euphorbia condylocarpa*: (Euphorbiaceae)—Euphorbia	0.2 mM HAuCl_4_ 1 mM PdCl_2_	1:1:1	50 °C with stirring	120	80	spherical	[64]
	*Cacumen platyclade:* (Cupressaceae)—Chinese Arborvitae Leaf and Twig	0.25 mM HAuCl_4_ 0.25 mM PdCl_2_	1:2:2	25 °C with stirring	120	7.4	spherical	[65]
	*Cacumen platyclade*: (Cupressaceae)—Chinese Arborvitae Leaf and Twig	5 mM HAuCl_4_ 5 mM Na_2_PdCl_4_	1:4:4	25 °C	30	47.8	flower-like core-shell	[66]
	*Citrus limon* L.: (Rutaceae)—Lemon	1 mM PdCl_2_ 1 mM HAuCl_4_·3H_2_O	1:9:9	25 °C	5	2–10	spherical	[67]
Au–Pt BNPs	*Asarum europaeum* L.:(Aristolochiaceae)—Asarabacca	1 mM HAuCl_4_ 1 mM K_2_PtCl_6_	1:1:1	80 °C	1440	5–6	spherical	[68]
	*Phragmites australis*: (Poaceae)—Common reed	1 mM HAuCl_4_ 1 mM K_2_PtCl_4_	1:5:5	85 °C	60	35.1 ± 2.71	flower-like core-shell	[69]
Pt–Pd BNPs	*Annona muricata* L.: (Annonaceae)—Sirsak	H_2_PtCl_6_·6H_2_O PdCl_2_, 0.25:0.75, 0.5:0.5, 0.75:0.25	1:1:3 1:1:1 1:3:1	100 °C with stirring	120	3.97–7.06	spherical	[70]
	*Dioscorea bulbifera* L.: (Dioscoreaceae)—Air potato	1 mM H_2_PtCl_6_·6H_2_O 1 mM PdCl_2_	1:1:1	100 °C	300	20–25	irregular shape	[71]
Cu–Ag BNPs	*Kigelia Africana*: (Bignoniaceae)—Sausage tree	1 mM CuCl_2_·H_2_O, 1 mM AgNO_3_	1:1:2	120 °C	60–360	10	spherical	[72]
	*Rheum emodi*: (Polygonaceae)—Rhubarb	10 mM AgNO_3_ 10 mM Cu(CH_3_COO)_2_	5:2:15	90 °C with stirring	180	40–50	spherical	[73]
ZnO–Ag BNPs	*Mirabilis jalapa*: (Nyctaginaceae)—Marvel of Peru	Zn(O_2_CCH_3_)_2_ (H_2_O)_2_ AgNO_3_ 0.5:0.5, 0.1:0.1, 0.5:0.1, 0.1:0.5 and 1:1 mM	1:1:1	60 ± 5 °C	120	19.3–67.4	spherical	[74]
Ag–Ni BNPs	*Senna occidentalis*: (Fabaceae)—Coffee senna	AgNO_3_ Ni(NO_3_)_2_·6H_2_O 1 mM:1 mM 1 mM:2 mM 2 mM:2 mM	1:5:5 1:5:10 1:10:10	700 °C with stirring	10	10.25 ± 4.19	pseudo-cubic	[75]

**Table 2:** The biological activity of BNPs and metallic NPs synthesised from natural extracts depending on the NPs size (BNPs—bimetallic nanoparticles, NPs—nanoparticles, IC—half-maximal inhibitory concentration, GAE—gallic acid equivalents, FRAP—ferric-reducing antioxidant power).

Plant Extract	NPs	Size	Experimental Findings	Ref.
*Terminalia chebula*	Ag–Pd BNPs	20 nm	48.45 ± 0.04 μg/mL (IC50–A549)	[47]
	Ag NPs	20–40 nm	18.09 ± 0.012 μg/mL (IC50–A549)	
	Pd NPs	20–40 nm	15.09 ± 0.012 μg/mL (IC50–A549)	
*Stigmaphyllon ovatum*	Au–Ag BNPs	14.9 nm	5.75 × 10^−16^ µM (IC50–HeLa)	[46]
	Au NPs	78 nm	0.0027 µM (IC50–HeLa)	
	Ag NPs	23.5 nm	9.1 × 10^−9^ µM (IC50–HeLa)	
*Gloriosa superba*	Au–Ag BNPs	10 nm	5.33 ± 0.33 mm (zone of inhibition—*E. coli*)	[15]
	Au NPs	20 nm	8.33 ± 0.33 mm (zone of inhibition—*E. coli*)	
	Ag NPs	20 nm	7.66 ± 0.33 mm (zone of inhibition—*E. coli*)	
*Vernonia mespilifolia*	Ag–Pt BNPs	35.5 ± 0.8 nm	44.1 mg GAE/g (FRAP)	[60]
	Ag NPs	33.4 ± 1.0 nm	18.5 mg GAE/g (FRAP)	
	Pt NPs	32 nm	16.5 mg GAE/g (FRAP)	
*Asarum europaeum*	Au–Pt BNPs	5–6 nm	excellent catalytic activity	[68]
	Au NPs	10 nm	-	
	Pt NPs	15 nm	-	
*Dioscorea bulbifera*	Pt–Pd BNPs	25 nm	74.25% anticancer activity (HeLa)	[71]
	Pt NPs	2.5 nm	12.6% anticancer activity (HeLa)	
	Pd NPs	10 nm	33.15% anticancer activity (HeLa)	
*Pulicaria undulata*	Au–Ag BNPs	5–12 nm	excellent catalytic activity	[58]
	Au NPs	10–20 nm	-	
	Ag NPs	10–20 nm	-	
*Crocus sativus*	Ag–Pt BNPs	36 nm	100.1 ± 0.32 μg/mL (zone of inhibition—*E. coli*)	[49]
	Ag NPs	32 nm	150.6 ± 0.41 μg/mL (zone of inhibition—*E. coli*)	
	Pt NPs	30 nm	154.2 ± 0.34 μg/mL (zone of inhibition—*E. coli*)	

**Table 3:** Biological activity of bimetallic nanoparticles (BNPs).

Plant Extract	BNPs Types	Size	Activity	Ref.
*Kigelia africana*	Cu–Ag	10 nm	Antimicrobial activities against Gram-negative bacteria (*Pseudomonas aeruginosa*, *Escherichia coli*, *Klebsiella pneumonia*) and Gram-positive bacteria (*Staphylococcus aureus*) and against the fungus (*Candida albicans*).	[72]
*Stigmaphyllon ovatum*	Ag–Au	14.9 nm	(In vitro) anticancer activity against HeLa cells.	[46]
*Guazuma ulmifolia*	Au–Ag	10–20 nm	Anticancer activity against HeLa cells. Antibacterial and antifungal activity. Catalytic activity against Congo red and 4-nitrophenol.	[142]
*Melia azedarach*	Au–Ag	10–20 nm	Antibacterial against *Bacillus cereus*, *Cronobacter sakazakii*, *Salmonella enterica*, *Escherichia coli*, *Listeria monocytogenes*, *Candida albicans.*	[143]
*Solidago canadensis*	Au–Ag	25.9 nm	Catalytic activity.	[14]
*Salvadora persica*	Ag–Ni	23.67 nm	Antioxidant activity.	[113]
*Dovyalis caffra*	Ag–Au	9–14 nm	Anticancer activity against the MCF7 cell line.	[144]
*Terminalia arjuna*	Cu–Ag	10–20 nm	The cytotoxic effect of biohybrid nanomaterials on different cell lines, MDA-MB-231, HeLa, SiHa, and He-G2 and non-toxic against Vero (normal epithelial cells). Antibacterial activity against bacterial strains *Escherichia coli*, *Staphylococcus aureus.*	[124]
*Crocus sativus*	Ag–Pt	36.0 nm	Antioxidant and antibacterial against Gram-positive and Gram-negative bacteria. Cytotoxic effect against pathogenic microbes and MCF-7 breast cancer cell line. Catalytic activity in the reduction of methyl orange.	[49]
*Madhuca longifolia*	Au–Ag	34–66 nm	Improving wound healing in vivo were examined in models of Swiss bee mice.	[57]
*Commelina nudiflora*	Au–Ag	20–80 nm	Bactericidal activity against selective oral pathogenic bacteria (*Escherichia coli*, *Pseudomonas aeruginosa*, *Bacillus subtilis*).	[56]
*Mirabilis jalapa*	ZnO–Ag	19.3–67.4 nm	Antioxidant, antibacterial, and antileishmanial activity.	[74]
*Arachis pintoi*	Ag–Mn	3.3 nm	Antibacterial activity against *Escherichia coli*, *Salmonella*, *Pseudomonas aeruginosa*, *Staphylococcus aureus*, and *Bacillus cereus.*	[145]
*Vitex negundo*	Ag–Cu	≈60 nm	Nanocomposites showed good antibacterial activity against Gram-positive and Gram-negative.	[146]
*Trifolium repens*	Au–Ag	15–20 nm Spherical	Electrochemical biosensor for the development of a respiration sensor for the detection of early gastric cancer.	[147]
*Pulicaria undulata*	Au–Ag	20–50 nm	Catalyst for the reduction of 4-nitrophenol to 4-aminophenol.	[58]
*Vernonia mespilifolia*	Ag–Pt	35.5 ± 0.8 nm, Spherical	Antimicrobial activity and antioxidant activity against DPPH and ABTS.	[60]
*Terminalia chebula*	Ag–Pt	20 nm Spherical	Anticancer activity against human lung cancer A549 cells. Antibacterial activity against MRSA and *Pseudomonas aeruginosa.*	[47]
*Asparagus racemosus*	Au–Ag	10–50 nm	Antibacterial and immunomodulatory activity.	[53]
*Citrus limon*	Au–Pd	2 nm	Larvicidal activity against mosquito larvae (*Anopheles stephensi* and *Aedes aegypti*) and non-target organs.	[67]
*Toddy palm*	Ag–Cu Cu–Zn	80–100 nm	Antibacterial activity against *Alcaligenes faecalis*, *Staphylococcus aureus*, *Citrobacter freundii*, *Klebsiella pneumoniae*, and *Clostridium perfringens.* Antitumor activity against nasopharyngeal cancer (KB) cells and Ehrlich ascites carcinoma (EAC) cell lines.	[108]
*Moringa oleifera*	Ag–Cu	87 nm	Antibacterial activity against *Staphylococcus aureus* and *Klebsiella pneumoniae.*	[148]
*Asian palmyra palm*	Ag–Co	-	Larvicidal activity against *Culex quinquefasciatus larvae.*	[141]
*Geum urbanum*	Ag–Au	50 nm	Evaluation of cytotoxicity.	[52]
*Cannabis sativa*	Au–Ag	48.79–83.44 nm	Antibacterial activity and antileishmanial activity against *Leishmania promastigote major.*	[119]
*Annona muricata*	Ag–Cu Ag–Zn	30 nm	Antidiabetic, antioxidant, and antibacterial activity.	[149]
*Artocarpus heterophyllus*	Au–Ag	15 nm	Significant antioxidant activity. Antibacterial activity for Gram-negative bacteria.	[55]
*Achras sapota*	Ag–Cu	≈20–40 nm Spherical	Investigated in vitro toxicity studies.	[150]
*Phoenix dactylifera*	Cu–Ag	26 nm	Catalytic activity for dye degradation (methylene blue) and antibacterial testing.	[135]
Quercetin and gallic acid	Ag–Se	30–35 nm	Antitumor effect on the proliferation/viability of Dalton lymphoma cell lines.	[89]

## Data Availability

Not applicable.
